# CXCL-16, IL-17, and bone morphogenetic protein 2 (BMP-2) are associated with overweight and obesity conditions in middle-aged and elderly women

**DOI:** 10.1186/s12979-017-0089-0

**Published:** 2017-03-11

**Authors:** Silvana Mara Turbino Luz Ribeiro, Laís Roquete Lopes, Guilherme de Paula Costa, Vivian Paulino Figueiredo, Deena Shrestha, Aline Priscila Batista, Roney Luiz de Carvalho Nicolato, Fernando Luiz Pereira de Oliveira, Juliana Assis Silva Gomes, Andre Talvani

**Affiliations:** 1 Department of Biological Sciences, Federal University of Ouro Preto, Ouro Preto, Minas Gerais Brazil; 2 Post-graduation Program in Biological Sciences/NUPEB, Federal University of Ouro Preto, Ouro Preto, Minas Gerais Brazil; 3 Post-graduation Program in Health and Nutrition, Federal University of Ouro Preto, Ouro Preto, Minas Gerais Brazil; 4 Post-graduation in Ecology of Tropical Biomas, Federal University of Ouro Preto, Ouro Preto, Minas Gerais Brazil; 5 Clinical Analyses Laboratory of the Pharmacy School, Federal University of Ouro Preto, Ouro Preto, Minas Gerais Brazil; 6 Department of Statistics, Federal University of Ouro Preto, Ouro Preto, Minas Gerais Brazil; 7 Laboratory of the Immunobiology of Inflammation, Federal University of Ouro Preto, Ouro Preto, Minas Gerais Brazil; 8 Department of Morphology, Federal University of Minas Gerais, Belo Horizonte, Minas Gerais Brazil

**Keywords:** Inflammation, Elderly, Overweight, Obesity, CXCL-16, BMP-2, IL-17

## Abstract

**Background:**

The current concept of overweight/obesity is most likely related to a combination of increased caloric intake and decreased energy expenditure. Widespread inflammation, associated with both conditions, appears to contribute to the development of some obesity-related comorbidities. Interventions that directly or indirectly target individuals at high risk of developing obesity have been largely proposed because of the increasing number of overweight/obese cases worldwide. The aim of the present study was to assess CXCL16, IL-17, and BMP-2 plasma factors in middle-aged and elderly women and relate them to an overweight or obese status. In total, 117 women were selected and grouped as eutrophic, overweight, and obese, according to anthropometric parameters. Analyses of anthropometric and circulating biochemical parameters were followed by plasma immunoassays for CXCL-16, IL-17, and BMP-2.

**Results:**

Plasma mediators increased in all overweight and obese individuals, with the exception of BMP-2 in the elderly group, whereas CXCL16 levels were shown to differentiate overweight and obese individuals. Overweight and/or obese middle-aged and elderly individuals presented with high LDL, triglycerides, and glycemia levels. Anthropometric parameters indicating increased-cardiovascular risk were positively correlated with CXCL-16, BMP-2, and IL-17 levels in overweight and obese middle-aged and elderly individuals.

**Conclusion:**

This study provides evidence that CXCL-16, IL-17, and BMP-2 are potential plasma indicators of inflammatory status in middle-aged and elderly women; therefore, further investigation of obesity-related comorbidities is recommended. CXCL16, in particular, could be a potential marker for middle-aged and elderly individuals transitioning from eutrophic to overweight body types, which represents an asymptomatic and dangerous condition.

## Background

Obesity is a worldwide epidemic and pathological condition resulting from contemporary lifestyles; it is characterized by lack of physical activity in addition to an energy-dense diet [1]. Adipose tissue was previously defined as a simple energy source used to manage energy flow in the body; however, it has taken on the integrative role of linking bioactive mediator release, homeostasis, and pathological conditions [2]. At least 24 typical inflammatory mediators such as leptin, resistin, IL-6, TNF, and chemokines, among others (e.g., C-reactive protein, haptoglobin, and amyloid A) are upregulated during obesity [3–5].

Natural ageing is currently associated with senescent immune remodeling, which occurs when innate barriers lose their abilities to restrict pathogen entry; in this condition, the internal organs become more vulnerable to metabolic disturbances resulting in a natural reduction in life expectancy [6, 7]. It is currently accepted that increased visceral omental, not abdominal subcutaneous adipose tissue, correlates the most with cardiovascular diseases, insulin resistance, and other obesity-related diseases [8, 9]. Overweight and obesity statuses often start early in child- or adulthood. These conditions become detrimental during senescence, when the human body requires balance to deal with age-associated changes. Accordingly, lifestyle and nutritional changes in infants and adults might prevent or postpone some adverse effects on the immune system and improve quality of life in elderly individuals. Both the inflammatory context and biological aging are crucial interrelated components of the understanding the association of clinical disturbances with overweight and obesity.

Based on the many potential biomarkers that have the capacity to predict obesity- and overweight-related diseases, we investigated the clinical role of three soluble mediators in human cardiac and metabolic diseases: (i) IL-17, a pro-inflammatory cytokine previously associated with obesity due to its role in the development of atherosclerosis, coronary syndromes, and glucose tolerance [10, 11], (ii) CXCL16, an interferon-gamma-regulated chemokine and scavenger receptor for oxidized low-density lipoprotein that is expressed in atherosclerotic lesions and described as a marker of acute coronary syndromes [12, 13], and finally, (iii) bone morphogenetic protein-2/BMP-2, a cytokine that promotes bone formation, but also is associated with potential adverse clinical effects including cyst-like bone formation, soft tissue swelling, and other anabolic activities in normal and osteoarthritic chondrocytes [14, 15]. Thus, the aim of the present study was to investigate the association between CXCL-16, BMP-2, and IL-17 plasma levels and overweight and obese conditions in middle-aged (35 to 55 years of age) and elderly (>60 years old) women to find possible targets to monitor early progression to obesity.

## Methods

### Study population

We performed this study using 46 healthy individuals (23 middle-aged and 23 elderly), 33 overweight individuals (20 middle-aged and 13 elderly), and 38 obese individuals (23 middle-aged and 15 elderly). All study participants were of the female sex and recruited at the UNIMED Inconfidentes, Ouro Preto, MG, Brazil. These women were undergoing a complete clinical examination and the following laboratory workup was performed: full blood count, free T4, TSH, glucose, cholesterol, VLDL, LDL, HDL, and triglycerides. Individuals with hypertension, diabetes, thyroid or renal disturbances, or any other cardiac or systemic diseases as well as those using steroidal drugs were excluded from this study. These conditions could prevent adequate interpretation of the association between obesity-related diseases and immune parameters. After blood collection and full clinical evaluation, all individuals were monitored weekly by health professionals (medical doctors, nutritionists, physiotherapist, and others) from UNIMED-Inconfidentes.

### Definition of anthropometric measures

All study participants had body mass, triceps skinfold, and arm, waist, and hip circumference measurements collected by a trained staff member, in accordance with standardized protocols [16, 17]. Body mass index was calculated as weight divided by squared height (kg/m^2^). The triceps skinfold was verified on the mid-line of the posterior surface of the arm (over the triceps muscle) at the level of the mid-point between the acromiale and the radiale. The subscapular skinfold was measured just below the angle of the scapula. Waist circumference was also measured at the midpoint between the lowest rib and the iliac crest.

Arm muscle circumference was the chosen indicator for elderly individuals, as the Third National Health and Nutrition Examination Survey (NHANES III) has presented reference data for this indicator [18]. All measurements were taken in a private room and conducted by an experienced nutritionist.

### Biochemical analysis

Blood samples (7 ml) were drawn using S-Monovette tubes (Sarstedt Ltda, Nümbrecht Germany) between 7:00 and 9:00 AM, from all study participants after 12–14 h of overnight fasting. All blood tests (cholesterol, VLDL, LDL, HDL, triglycerides, and glycemia) were performed using conventional enzymatic methods at the certified Clinical Analysis Laboratory from the Pharmacy School at Universidade Federal de Ouro Preto, MG, Brazil, on the day of blood collection.

### Immunoassays for inflammatory mediators

Circulating levels of the inflammatory mediators, IL-17, CXCL16, and BMP-2, were detected in plasma samples that were previously stored at −80°C. Briefly, flat-bottom 96-well microtiter plates (Nunc) were coated with 100 μl/well of appropriate monoclonal antibodies for 18 h at 4°C and then washed with PBS buffer (pH 7.4) containing 0.05% tween-20 (wash buffer). Non-specific binding sites were blocked with 200 μl/well of 1% bovine serum albumin in PBS. Plates were washed and 100 μl/well of sample was added, which was followed by incubation for 18 h at 4°C. In addition, 100 μl/well of the appropriate biotinylated detection antibody, diluted in blocking buffer containing 0.05% tween-20, was added and samples were incubated for 1 h at 24°C. Streptavidin-horseradish peroxidase was added and after subsequent incubation and washing, 100 μl/well of the chromogen substrate o-phenylenediamine (Sigma-Aldrich Brasil Ltda, SP, Brazil), diluted in 0.03 M citrate buffer containing 0.02% of H_2_O_2_ was added for 30 min, which was followed by incubation in the dark at room temperature. The reaction was stopped through the addition of 50 μl/well of 1 M H_2_SO_4_ solution and plates were read at 492 nm in a spectrophotometer (Emax_Molecular Devices, Sunnyvale, CA, USA). All samples were simultaneously measured using Peprotech ELISA kits (Ribeirão Preto, SP, Brazil) in triplicate.

### Statistical analysis

Graph Pad InStat and Prism statistical programs were used for analysis (GraphPad, San Diego, CA, USA). In cases of normal distribution, the independent *t* test was used, whereas the non-parametric Mann-Whitney test was used for non-Gaussian sample distributions. Correlations were verified using a Spearman test and results were confirmed by performing a linear regression test. Data were considered statistically significant when *p* < 0.05.

## Results

### Anthropometric characterization of subjects

Our study population consisted of 63 middle-aged (mean age of 40.9 years) and 54 elderly (mean age of 68.9 years) women, categorized according anthropometric parameters as eutrophic, overweight, and obese (Table 1). All anthropometric parameters such as body mass, triceps skinfold, arm circumference, arm muscle circumference, and waist and hip measurements were elevated in overweight or obese middle-aged and elderly individuals when compared to eutrophic individuals. Body mass index similarly increased with weight gain in both age groups, but the waist-to-hip ratio only increased in obese middle-aged individuals, and not in overweight or obese elderly individuals (data not shown).

**Table 1:** Baseline anthropometric parameters of the eutrophic, overweight and obesity subjects

	Eutrophic	Overweight	Obesity
	Adult (*n* = 23) / Elderly (*n* = 23)	Adult (*n* = 20) / Elderly (*n* = 13)	Adult (*n* = 23) / Elderly (*n* = 15)
Age	40,0 ± 1,2 / 71,5 ± 1,4	40,1 ± 1,5 / 64,0 ± 2,0	42,6 ± 1,5 / 71,4 ± 2,1
Body mass (kg)	57,3 ± 1,6 / 59,1 ± 1,6	68,8 ± 1,0* / 70,7 ± 2,1*	86,8 ± 2,6*# / 80,3 ± 1,6*A#
BMI (Kg/m2)	21,8 ± 0,4 / 23,6 ± 0,4	27,2 ± 0,2* / 28,26 ± 0,3*	34,0 ± 0,6*# / 33,2 ± 0,5*#
TSF (mm)	17,0 ± 1,1 / 18,4 ± 1,4	25,8 ± 1,3* / 24,0 ± 1,4	32,2 ± 1,5*# / 28,3 ± 1,5*
AC (cm)	27,1 ± 0,5 / 29,2 ± 0,5	31,3 ± 0,4* / 33,2 ± 0,6*	36,4 ± 0,7*# / 35,5 ± 0,6*
AMC (cm)	21,7 ± 0,5 / 23,4 ± 0,3	23,2 ± 0,4 / 25,7 ± 0,5*A	26,0 ± 0,5*# / 26,8 ± 0,5*
Waist (cm)	82,1 ± 1,6 / 87,1 ± 1,9	91,5 ± 1,5* / 101,1 ± 2,2*A	107,7 ± 2,4*# / 102,9 ± 1,7*

Values are shown as the mean ± SEM. *BMI* body mass index, *TSF* Triceps skinfold, *AC* arm circumference, *AMC* arm muscle circumference. * *p* < 0.01 when eutrophic groups were compared with their respective chronological overweight (OW) or obesity (OB) groups; ^A^ *p* < 0.05 when adult and elderly were compared among in terms of eutrophic, OW or OB; # *P* < 0.01 when OW were compared with OB inside the same chronological groups

An apparent reduction in immune and physiological activity in the senescent body was observed under natural conditions and without the presence of disease. Overweight and obese middle-aged individuals presented with anthropometric parameters that were worse than those in elderly eutrophic individuals, based on all analyzed parameters, except for arm muscle circumference. In addition, overweight and obese conditions were associated with similar anthropometric parameters, in both groups of women, with a minimum age difference of 30 years.

### Cholesterol and glucose parameters in overweight and obese women

Blood biochemical parameters are shown in Table 2. VLDL cholesterol and glycemia levels were higher in eutrophic elderly individuals than in eutrophic middle-aged individuals.

**Table 2:** Biochemical parameters in eutrophic, overweight and obesity women

Eutrophic	Overweight	Obesity
Adult (*n* = 23) / Elderly (*n* = 23)	Adult (*n* = 20) / Elderly (*n* = 13)	Adult (*n* = 23) / Elderly (*n* = 15)
Cholesterol (mg/dL) 157,8 ± 6,5 / 211,0 ± 12,0^A^	194,0 ± 9,3* / 193,8 ± 13,0	217,0 ± 8,4* “/ 195,9 ± 11,0
VLDL (mg/dL) 13,4 ± 1,0 / 18,8 ± 2,1^A^	17,9 ± 2,8 / 27,05 ± 1,8^A^ *	22,0 ± 2,3* “/ 28,1 ± 2,9^A^ *
LDL (mg/dL) 125,9 ± 6,1 / 133,0 ± 11,0^A^	119,9 ± 8,5 / 121,3 ± 12,1	143,2 ± 7,7“/ 125,8 ± 11,6
HDL (mg/dL) 58,3 ± 1,8 / 55,7 ± 2,3	53,6 ± 2,7 / 54,7 ± 3.2	51,5 ± 2,2“/ 46,5 ± 2,1“
Triglycerides (mg/dL) 67,5 ± 5,1 / 86,9 ± 10,6^A^	89,5 ± 14,0 / 85,6 ± 9,1	111,4 ± 11,5“/ 115,1 ± 10,9“
Glycemia (mg/dL) 91,0 ± 1,9 / 103,2 ± 9,3^A^	91,3 ± 1,8 / 119,6 ± 8,8^A^ *	104,4 ± 4,7* “/ 117,5 ± 11,9^A^ *

Values are shown as the mean ± SEM. *VLDL* very low density lipoproteins, *LDL* low density lipoproteins, *HDL* High density lipoproteins. Statistical differences are represented being that (*) *p* < 0.05 when the OW or OB were compared with the eutrophic individuals in the same age group; (^Δ^) *p* < 0.05 when adult and elderly were compared in the same anthropometric group, (^∞^) when OB is different from OW in the same age group

However, when anthropometric parameters were taken into consideration for this biochemical analysis, overweight and/or obese individuals had total cholesterol, VDLL cholesterol, and glycemia levels that were higher than those in the respective age-matched eutrophic control groups. When we compared overweight and obese statuses, obese middle-aged individuals had higher total cholesterol, VLDL, LDL, HDL, triglycerides, and glycemia levels, whereas only HDL and triglycerides were different in the obese elderly group.

Interestingly, soluble CXCL16, but not IL-17 and BMP-2, was positively correlated with glycemia in middle-aged (*r* = 0.734, *p* < 0.01) and elderly (*r* = 0.604, *p* < 0.05) individuals, as well as with VLDL cholesterol in middle-aged (*r* = 0.623, *p* < 0.05) and elderly (*r* = 0.546, *p* < 0.05) individuals.

### Plasma indicators of overweight and obesity

**Fig. 1:**
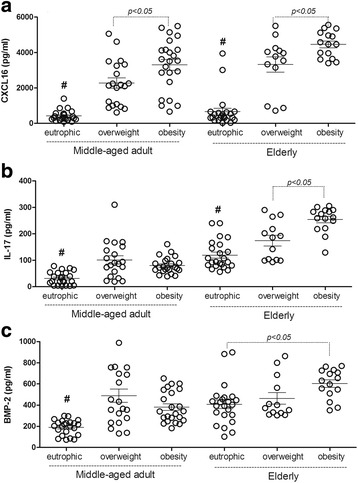
Concentrations of the CXCL16, IL17 and BMP-2 plasma in middle-aged adult and elderly women. The concentrations (pg/ml) of chemokine CXCL16 plasma (**a**) The bone morphogenetic protein (BMP-2) (**b**) the inteleukin-17 (IL-17) (**c**) from middle-aged adult and elderly women presenting eutrophic, overweight and obesity conditions were measured through ELISA. The individual results were plotted in the graphic through mean ± SEM. # *P* < 0.05 when all groups were different from the eutrophic middle-aged adult or elderly subjects

Plasma levels of the inflammatory chemokine CXCL16 (Fig. 1a) were markedly elevated in overweight and obese middle-aged and elderly individuals when compared to that in eutrophic individuals In addition, CXCL16 was capable of distinguishing overweight and obese individuals in the two age-related groups. CXCL16 production in overweight or obese middle-aged individuals was higher than that observed in 60-year old eutrophic individuals.

IL-17 (Fig. 1b) production was high in all overweight and obese individuals. However, obese elderly individuals presented with higher cytokine levels than overweight individuals of the same age group. In terms of IL-17 production, there were no differences between overweight and obese middle-aged and eutrophic elderly individuals.

BMP-2 (Fig. 1c) was higher in all overweight and obese middle-aged individuals, similar to that observed with the Il-17 pattern, but there was no difference observed between eutrophic and overweight elderly individuals. Increased production of this factor was observed in eutrophic and obese elderly individuals, suggesting natural degeneration of the bones.

### Correlations among CXCL16, IL-17, and BMP-2 and anthropometric parameters

We assumed that body mass index, triceps skinfold, arm muscle circumference, and hip and waist circumferences are variables used to predict obesity-related comorbidities. The correlations among these anthropometric parameters and plasma CXCL16, BMP-2, and IL-17 levels are shown in Table 3. Accordingly, CXCL16 showed the highest correlation with body mass index, triceps skinfold, and waist and hip circumference in middle-aged and elderly individuals, with Pearson’s correlation coefficients (r) > 0.5.

**Table 3:** Correlation among CXCL16, IL-17 and BMP-2 and anthropometric parameters

ADULTS	BMI	TSF	AMC	WAIST	HIP	WHR
CXCL16	*R* = 0.798*	*R* = 0,552*	*R* = 0.119	*R* = 0.860*	*R* = 0.555*	*R* = 0.2081
	*p* = 0.023	*p* = 0.5521	*p* = 0.1981	*p* = 0.016	*p* = 0.050	*p* = 0 . 1 9 8 1
IL-17	*R* = 0.188*	*R* = 0.354	*R* = 0.371*	*R* = 0.341	*R* = 0.556*	*R* = 0.221*
	*p* = 0.047	*p* = 0.850	*p* = 0.010	*p* = 0.117	*p* = 0.050	*p* = 0.014
BMP-2	*R* = 0.1629	*R* = 0.0935	*R* = 0.1778	*R* = 0.2423	*R* = 0.1518	*R* = 0.1778
	*p* = 0.1034	*p* = 0,3523	*p* = 0.0752	*p* = 0.0146	*p* = 0.1295	*p* = 0.075
ELDERLY	BMI	TSF	AMC	WAIST	HIP	WHR
CXCL16	*R* = 0.798*	*R* = 0.552*	*R* = 0.198	*R* = 0.860*	*R* = 0.555*	*R* = 0.198
	*p* = 0.020	*p* = 0.050	*p* = 0.119	*p* = 0.016	*p* = 0.050	*p* = 0 . 1 1 0
IL-17	*R* = 0.184*	*R* = 0.332	*R* = 0.227*	*R* = 0.146	*R* = 0.542*	*R* = 0.371*
	*p* = 0.030	*p* = 0.090	*p* = 0.014	*p* = 0.110	*p* = 0.050	*p* = 0.020
BMP-2	*R* = 0.179*	R = 0.352	*R* = 0.187*	*R* = 0.242*	*R* = 0.251	*R* = 0.188*
	*p* = 0.050	*p* = 0,090	*p* = 0.050	*p* = 0.014	*p* = 0.129	*p* = 0.050

*BMI* body mass index, *TSF* triceps skinfold, *AMC* arm muscle circumference, *WHR* waist-to-hip ratio

* positive correlation with *p* < 0.05

Finally, IL-17 plasma levels were also positively correlated with body mass index, arm muscle circumference, and hip and waist circumferences in both age-related groups, whereas BMP-2 levels were only correlated with these anthropometric parameters in the elderly group.

## Discussion

Results presented herein highlight the potential for CXCL16, IL-17, and BMP-2 plasma levels to be used as marker to discriminate overweight and obese individuals in both age groups. In particular, production of the chemokine CXCL16 was markedly high in the overweight and obese groups, and this correlated with anthropometric parameters that are considered comorbidity predictors.

The identification of biomarkers to predict obesity has become in important research area within the basic and clinical sciences, owing to the ability of these factors to predict cardiovascular and metabolic disturbances; this allows for proper treatment and lifestyle changes in individuals [19]. Unfortunately, the underlying complexity of biological pathway interactions demands further research before any of these potential biomarkers can be accurately used for diagnostic purposes. Many cellular inflammatory mediators come from white fat tissues, and they are often overproduced because of increased adiposity. In contrast, other regulatory factors or mediators of insulin sensitivity, such as adiponectin, are found in smaller amounts in similar conditions [20]. This imbalance between adipocyte products and other inflammatory mediators, caused by overweight and obese conditions might promote obesity-linked metabolic and cardiovascular disorders [1, 9]. Therefore, investigating biomarkers in overweight individuals should be a more common procedure in research and clinic. Ordinary individuals naturally gain a small amount of weight within a certain period-of-time; however, continuous chronic weight gain becomes problematic.

Interestingly, CXCL16 was shown to have a strong and exclusive association with overweight and obese conditions, regardless of subject age. Elderly women with higher VLDL and glycemia typically had higher levels of circulating CXCL16; these high circulating levels have previously been associated with the development of plaque deposits, artery walls, and/or diabetes [18, 20, 21]. Anthropometric parameters such as body mass index, triceps skinfold, and waist and hip circumference were positively correlated with CXCL16 levels in middle-aged and elderly women. CXCL16 is expressed in macrophages and aortic muscle cells. Expression increases in response to inflammatory stimuli and this enhances LDL uptake in favor of foam cell formation in human atherosclerotic plaques [11, 13]. Some chemokines have been described as effective adipokines and as biomarkers of obese conditions because of their inflammatory profile, which is associated with weight gain and adipose tissue accumulation, and the fact that their receptors are overexpressed in subcutaneous and visceral adipose tissues of obese individuals [22]. Previous studies have indicated an association between CXCL16 and obesity in C57BL6 mice [23] and coronary comorbidities in humans [24–26]. By assuming that obesity predisposes individuals to coronary and metabolic diseases, the present study included overweight and obese women. Regardless of this, high plasma levels of CXCL16 were observed in all middle-aged and elderly individuals presenting with an anthropometric status above overweight.

Overweight or obese individuals were expected to have increased adipocyte numbers, resulting from the recruitment of pluripotent stem cells to the vascular stroma of adipose tissue [27]. In our study, BMP-2 was investigated and was shown to be clearly elevated in overweight and obese middle-aged and elderly individuals. Regarding anthropometric parameters, this factor was positively correlated with body mass index, arm muscle circumference, and waist and waist-to-hip ratio in elderly women. These parameters are indicative of overweight or obese conditions, reinforcing the potential role BMP-2 to categorize individuals according to metabolic status. BMP-2 has been shown to promote osteogenic differentiation and adipogenesis in bone marrow stromal cells [28], and it could be partially responsible for increased obesity. A recent study suggested that increased demand to store excessive energy promotes BMP-2 expression and that adipocytes contribute to this phenomenon, due to energy storage in visceral and subcutaneous adipose tissues [29]. The present study reinforces such findings, since increased BMP-2 levels were observed in overweight and obese women and were associated with anthropometric parameters that indicate adipose tissue accumulation.

Unlike BMP-2, IL-17 has emerged as a negative regulator of adipogenesis and glucose metabolism in an experimental model. This cytokine acts in a complex network of inflammatory mediators that links adipose tissue to inflammatory cells such as neutrophils and adipocytes [10, 30, 31]. Indeed, excessive production of IL-17 during the initial stage of obesity could have a different effect on maintaining a prolonged inflammatory response and on associated long-term consequences. IL-17 was remarkably high in elderly individuals, and particularly in the obese and overweight middle-aged individuals; here, higher levels of VLDL and glycemia were observed. In addition, IL-17 was also positively correlated with classical anthropometric parameters (body mass index, arm muscle circumference, and hip and waist-to-hip ratio). These parameters have been described as anthropometric markers of poor prognosis in metabolic and cardiovascular diseases [19]. They have also been shown to contribute to the release of other inflammatory cytokines such as IL-6, IFN-gamma, and TNF [10], which reinforces the potential role of IL-17 in obesity-related diseases.

Our data reinforce the fact that obese individuals (middle-aged and elderly) present with higher cholesterol fractions, triglycerides, and glycemia, when compared to those parameters in eutrophic individuals, in accordance with the previous studies [1]. However, these overweight middle-aged individuals must receive follow-up care from health professionals when considering the relevance of visceral obesity, alongside soluble biomarkers. Nonetheless, integrated intervention programs based on weight reduction are demanding, since the immune conditions (systemic inflammation) in these patients are similar to those in elderly individuals. This might indicate the presence of accelerated damage due to the production of inflammatory mediators (e.g. CXCL-16, IL-17, and BMP-2), since high levels of these circulating factors clearly represent a risk for cardiovascular disease, cancer, and metabolic disturbances [12, 19, 25]. Preventing and reducing overweight and obese conditions depends on medical interventions, in addition to individual lifestyle changes; however, further research to motivate behavioral changes is required.

## Conclusion

The present study provides complementary information regarding BMP-2, IL-17, and particularly CXCL-16, and their potential to be used as soluble markers capable of differentiating overweight and obese statuses in middle-aged and elderly women. These biomarkers were shown to be correlated with anthropometric (body mass index, triceps skinfold, arm muscle circumference, and hip and waist circumference) and biochemical (VLDL and glycemia) parameters. These factors might alter the inflammation axis observed in overweight and obese individuals, as well as in related disorders such as atherosclerosis, insulin resistance, and vascular damage. However, their clinical application needs to be investigated further in prospective population studies since its application in the clinical screening of overweight could help early in the preventive management of diseases associated with obesity.

## Abbreviations

BMP-2: Bone morphogenetic protein-2
CXCL-16: CXC16 ligand
HDL: High density lipoprotein
IL-17: Interleukin-17
IL-6: Interleukin-6
LDL: Low density lipoprotein
T4: Thyroxine
TNF: Tumor necrosis factor
TSH: Thyroid-stimulating hormone
VLDL: Very low density lipoprotein
